# Loss of MT1-MMP in Alveolar Epithelial Cells Exacerbates Pulmonary Fibrosis

**DOI:** 10.3390/ijms22062923

**Published:** 2021-03-13

**Authors:** Luis Placido, Yair Romero, Mariel Maldonado, Fernanda Toscano-Marquez, Remedios Ramírez, Jazmín Calyeca, Ana L. Mora, Moisés Selman, Annie Pardo

**Affiliations:** 1 Facultad de Ciencias, Universidad Nacional Autónoma de México, Mexico City 04510, Mexico; clow_636@hotmail.com (L.P.); yair@ciencias.unam.mx (Y.R.); toscano.fm@gmail.com (F.T.-M.); remediosra@yahoo.com.mx (R.R.); calyecajaz@gmail.com (J.C.); 2 Instituto Nacional de Enfermedades Respiratorias “Ismael Cosío Villegas”, Mexico City 14080, Mexico; vaqueis@gmail.com (M.M.); mselmanl@yahoo.com.mx (M.S.); 3 Division of Pulmonary Allergy and Critical Care Medicine, Department of Medicine, University of Pittsburgh, Pittsburgh, PA 15260, USA; anamora@pitt.edu

**Keywords:** Matrix metalloproteinases, MMP, lung fibrosis, IPF

## Abstract

Idiopathic pulmonary fibrosis (IPF) is a lethal age-related lung disease whose pathogenesis involves an aberrant response of alveolar epithelial cells (AEC). Activated epithelial cells secrete mediators that participate in the activation of fibroblasts and the excessive deposition of extracellular matrix proteins. Previous studies indicate that matrix metalloproteinase 14 (MMP14) is increased in the lung epithelium in patients with IPF, however, the role of this membrane-type matrix metalloproteinase has not been elucidated. In this study, the role of Mmp14 was explored in experimental lung fibrosis induced with bleomycin in a conditional mouse model of lung epithelial MMP14-specific genetic deletion. Our results show that epithelial Mmp14 deficiency in mice increases the severity and extension of fibrotic injury and affects the resolution of the lesions. Gain-and loss-of-function experiments with human epithelial cell line A549 demonstrated that cells with a deficiency of MMP14 exhibited increased senescence-associated markers. Moreover, conditioned medium from these cells increased fibroblast expression of fibrotic molecules. These findings suggest a new anti-fibrotic mechanism of MMP14 associated with anti-senescent activity, and consequently, its absence results in impaired lung repair. Increased MMP14 in IPF may represent an anti-fibrotic mechanism that is overwhelmed by the strong profibrotic microenvironment that characterizes this disease.

## 1. Introduction

Idiopathic pulmonary fibrosis (IPF) is an age-related chronic, progressive, and usually lethal interstitial lung disease of unknown etiology. A growing body of evidence indicates that the disease represents an epithelial-driven disorder whereby an aberrantly activated lung epithelium produces mediators for fibroblast migration, proliferation, and differentiation into active myofibroblasts that secrete exaggerated molecules of extracellular matrix (ECM) provoking a progressive and irreversible loss of lung structure and function [1,2,3].

Several matrix metalloproteases (MMPs) expressed by the lung epithelium play a critical pathophysiological role in IPF including MMP-1, MMP-7, MMP 19, and MMP-28 [4,5,6,7,8]. However, different studies in experimental lung fibrosis have revealed that some MMPs may have a profibrotic effect while others show an antifibrotic function [4]. For example, transgenic mice null of Mmp3, 7, 8, and 28 promote fibrosis [5,8,9,10] while Mmp13 and 19 have shown the opposite effect [7,11]. 

MMP14, also called membrane type-1 matrix metalloproteinase (MT1-MMP), is a membrane-associated MMP that was originally discovered as a proMMP-2 activator, expressed on the surface of invasive cancer cells [12]. Extensive evidence has demonstrated that it is implicated in ECM remodeling since it degrades a variety of their components including fibrillar collagens [13]. Previous studies in our lab showed that MT1-MMP (Mmp14) is increased in bleomycin-induced lung fibrosis and in IPF the enzyme is localized mainly in the alveolar epithelium [14,15]. However, the specific role of MT1-MMP in the disease is unknown. Usually, the role of MMPs in cell function has been strengthened by the analysis of experimental models with transgenic mice lacking the different enzymes; however, loss of mouse MT1-MMP (Mmp14 null mice) results in premature death 3 weeks after birth [16]. In this context, in this study we developed a conditional Mmp14 null mouse specific for type2 alveolar epithelial cells (AEC2) to evaluate the role of this membrane-type MMP (MT1-MMP) in experimental lung fibrosis as well as gain- and loss-of-function experiments with A549 epithelial cells to unravel possible mechanisms. Our results showed that AEC2-specific MT1-MMP-deficiency worsens bleomycin-induced lung fibrosis in mice and delay resolution. Epithelial silencing of this enzyme in vitro was associated with the induction of senescence.

## 2. Results

### 2.1. Matrix Metalloprotease 14 (MMP14) is Expressed Primarily in the Epithelium of Idiopathic Pulmonary Fibrosis (IPF) Patients 

**Figure 1:**
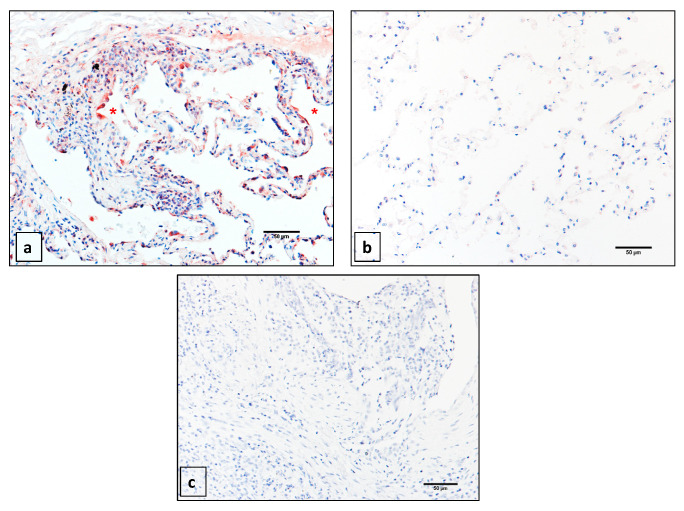
Immunohistochemistry of matrix metalloprotease 14 (MMP14) in idiopathic pulmonary fibrosis (IPF) and control tissues. (**a**) IPF lung showing immunoreactive protein primarily located in epithelial cells (*). (**b**) MMP14 in lung tissue from a normal donor. (**c**) Immunostaining control without primary antibody in lung tissue from IPF. Scale bar 50 μm.

To confirm our previous observations, we performed immunohistochemical analysis to determine the expression pattern of MMP14 in IPF and normal lung tissue sections by using specific antibodies. As illustrated in Figure 1a, strong MMP14 expression was observed in IPF, which was largely localized to the alveolar epithelium, mainly in hyperplastic cuboidal type 2 pneumocytes. The immunoreactive protein was also noticed in some fibroblast like cells.

By contrast, in sections from the control lung, the immunoreactive MMP14 protein was undetectable (Figure 1b). No specific signal was observed when the specific antibody was omitted (Figure 1c). These findings confirm the overexpression of MMP14 in alveolar epithelia of IPF lungs, suggesting that this enzyme may have a specific role in this cell type during fibrosis. 

### 2.2. Generation of a Novel Conditional Mouse Model of Lung Epithelial MMP14-Specific Genetic Deletion

**Figure 2:**
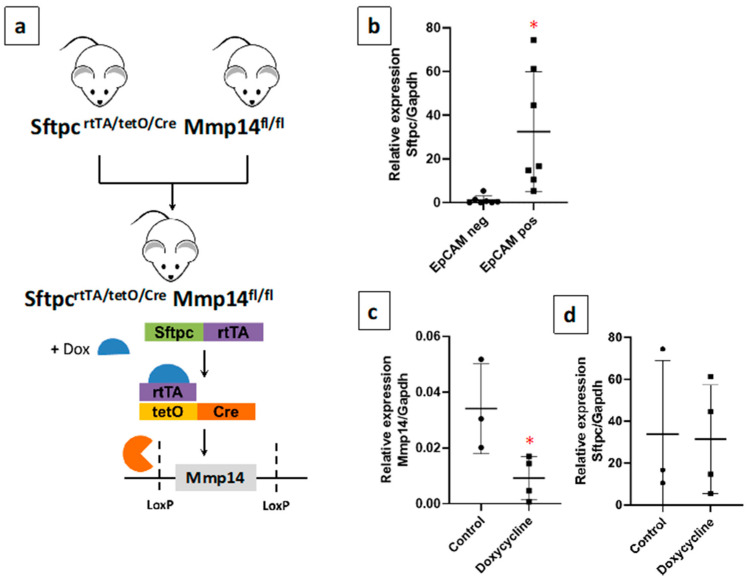
Mmp14 deletion in type 2 alveolar epithelial cells. (**a**) Schematic diagram demonstrating cell-specific Mmp14 deletion. Doxycycline-inducible Sftpc^rtTA/TetO/Cre^ mice were crossed with Mmp14^fl/fl^ mice. (**b**) Sftpc expression in isolated cells and selected by EpCAM microbeads. (**c**,**d**) Mmp14 and Sftpc expression in the positive EpCAM fraction from conditional KO mouse with/without doxycycline treatment. * p < 0.05 two-tailed *t*-student test. *n* ≥ 3.

To investigate the role of MMP14 expressed in the alveolar epithelium in experimental lung fibrosis, Sftpc^rtTA/tetO/Cre^ and Mmp14^fl/fl^ mice were used to generate a conditional knockout (cKO) Sftpc-Mmp14-cKO mice carrying a doxycycline-inducible Cre recombinase. In the Tet-ON system, doxycycline administration allows binding of the rtTA protein to the tetO sequence, which activates Cre expression. The tissue specificity is provided by the Sftpc promoter that drives rtTA protein expression (Figure 2a). Under basal conditions, Sftpc-Mmp14-cKO mice did not show differences in phenotype, lifespan, or reproductive capacity compared to wild-type (WT) mice. 

Isolated type 2 AECs were used to verify the loss of Mmp14 after doxycycline treatment. For this purpose, mice lungs were treated with dispase to obtain a cell suspension and the epithelial cell fraction was separated with magnetic beads using the Epithelial Cell Adhesion Molecule (EpCAM) antibody. By quantitative polymerase chain reaction (qPCR), surfactant protein C (Sftpc) expression was used as a marker of alveolar type 2 cells after EpCAM selection (Figure 2b). Treatment with doxycycline led to a significant reduction (*p* = 0.04) of Mmp14 mRNA in type 2 AECs isolated from Sftpc-Mmp14-cKO mice compared with those who did not receive doxycycline (Figure 2c), demonstrating that Mmp14 deficiency occurs in epithelial cells after doxycycline treatment. Additionally, we tested whether Sftpc expression changes by doxycycline or by the deletion of Mmp14 in EpCam positive cells. As shown in Figure 2d, there was no difference, indicating that in both conditions there was approximately the same amount of AEC2.

### 2.3. Epithelial Deletion of Mmp14 Exacerbates and Delays Resolution of Bleomycin-Induced Lung Fibrosis 

**Figure 3:**
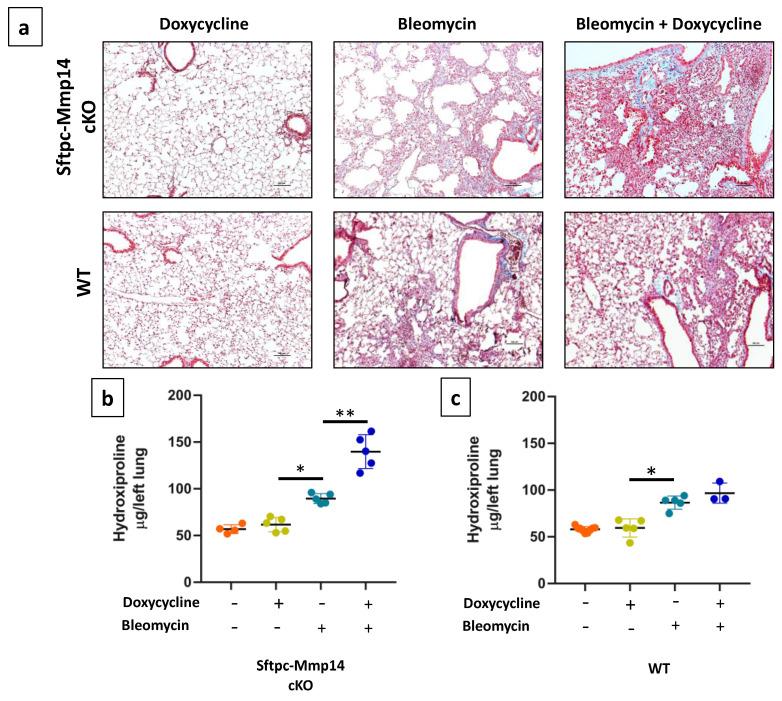
Lung injury induced with bleomycin in Sftpc-Mmp14 cKO and wild-type (WT) mice sacrificed at 21 days (fibrotic phase). (**a**) Representative micrograph stained with Masson’s trichrome (blue) for collagen deposition throughout the lungs of Sftpc-Mmp14 cKO and WT mice in the absence or presence of doxycycline, Scale bar 100 μm; (**b**,**c**) Hydroxyproline content in Sftpc-Mmp14 cKO (**b**) and WT mice (**c**) after bleomycin treatment in the absence or presence of doxycycline. * *p* < 0.05, ** *p* < 0.01 by one-way analysis of variance (ANOVA) with Tukey post-hoc *n* ≥ 3. Scale 100 μ.

To identify the role of MMP14 in the development of lung fibrosis, Sftpc-Mmp14-cKO and control mice were treated with a single intratracheal injection of bleomycin and evaluated at two time-points, 21 and 120 days that represents the fibrosis and resolution phases. At 21 days after instillation, Sftpc-Mmp14-cKO mice that received bleomycin and doxycycline, thus silencing Mmp14, displayed remarkable lung lesions, and more extensive accumulation of collagen fibers as indicated by Masson trichrome staining, compared with Mmp-14 deficient mice or WT littermates that received only bleomycin (Figure 3a). Semi-quantitative analysis of the extent of lesions showed a significant increase in the bleomycin-injured lungs of Mmp-14 deficient mice compared with WT (50 ± 10% versus 26.7 ± 15.3%, *p* < 0.05). Moreover, mice lacking Mmp14 showed severely distorted lung architecture.

Using a quantitative method, we evaluated fibrosis biochemically by measuring hydroxyproline content (OH-Pro), as a marker for collagen accumulation. First, we examined OH-Pro concentration under basal conditions. Sftpc-Mmp14-cKO mice with/without doxycycline exhibited similar levels of OH-Pro than C57BL/6 WT mice with/without doxycycline, suggesting that the genetic background of transgenic mice or doxycycline treatment per se does not affect lung collagen concentration (Figure 3b,c). Likewise, without doxycycline, both Sftpc-Mmp14-cKO and control mice showed a similar bleomycin-induced increase in the levels of OH-Pro. By contrast, after doxycycline and the consequent silencing of Mmp14, OH-Pro content was significantly higher in Mmp14-deficient mice compared to control mice (139 ± 18 vs. 89 ± 5 μg/lung *p* < 0.01) (Figure 3b,c). 

**Figure 4:**
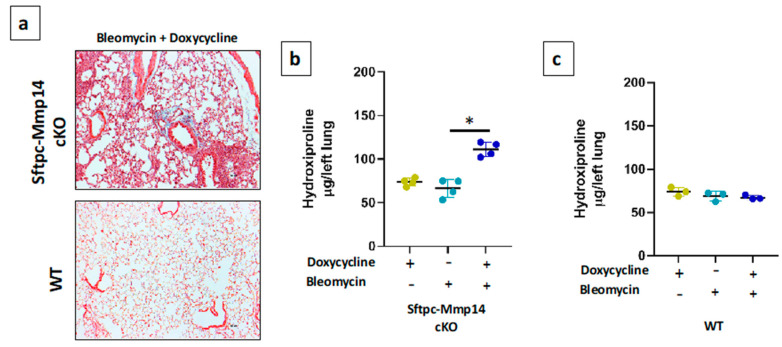
Lung injury induced with bleomycin in Sftpc-Mmp14 cKO and WT mice sacrificed at 120 days (resolution phase). (**a**) Representative micrograph stained with Masson’s trichrome (blue) for collagen deposition throughout the lungs of Sftpc-Mmp14 cKO and WT mice in presence of doxycycline. Hydroxyproline content in (**b**) Sftpc-Mmp14 cKO and in (**c**) WT mice, after bleomycin treatment in the absence or presence of doxycycline. * *p* < 0.05 by one-way ANOVA with Tukey post-test *n* ≥ 3. Scale bar 100 μm.

At 120 days after instillation, Sftpc-Mmp14 cKO mice showed an incomplete histopathological resolution (Figure 4a), with only a partial regression of the extent of the lesion (25 ± 12% versus 7.5 ± 5% in control mice). Likewise, the levels of OH-Pro remain higher while in control mice were reduced to almost normal levels (111 ± 8 vs. 66 ± 10 μg/lung *p* < 0.01; Figure 4 b,c), indicating that Mmp14 deficiency in AEC2 also affects the resolution of fibrosis. 

### 2.4. Epithelial MMP14 Deficiency Promotes Senescence In Vitro 

**Figure 5:**
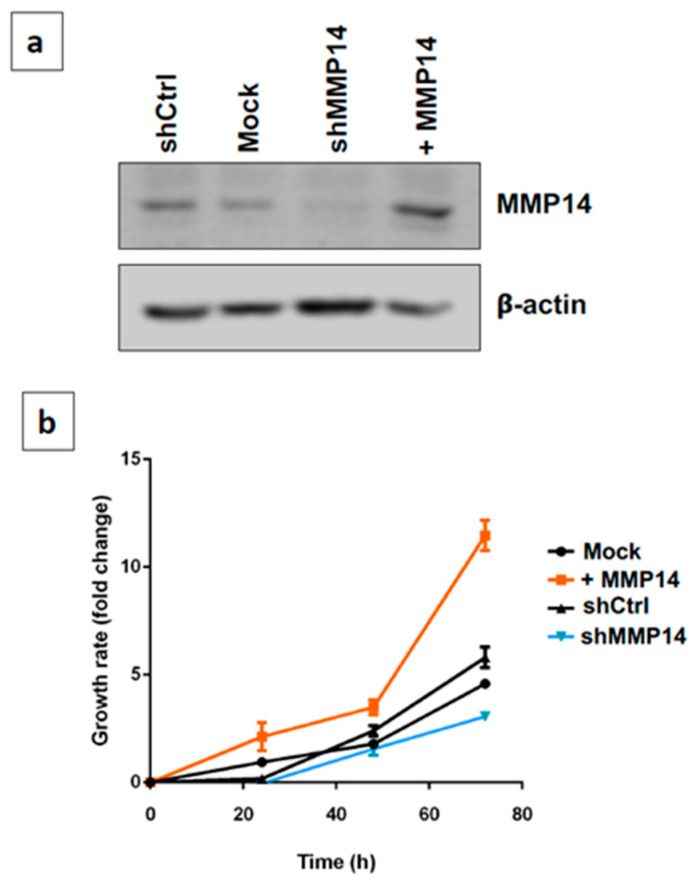
Effect of gain and loss of MMP14 on growth rate in A549 epithelial cells. (**a**) Western blot showing the attenuation of MMP14 in silenced cells and its overexpression in MMP14 transfected cells. (**b**) Cell overexpressing MMP14 showed increased growth rate, while the silencing decreases growth rate (mean ± standard deviation (SD)). Graphics represent one of three independent experiments by triplicate.

Epithelial cell senescence is one of the mechanisms that increases fibrogenic activity in vitro and aggravates lung fibrosis in vivo. Using a gain/loss of function approach we examined the effect of MMP14 on lung epithelial cell proliferation. For this purpose, both the 5 shRNAMT1-MMP (shMMP14) silencing vector and the pCMV6-AC-GFP + MMP14 (+MMP14) overexpression vector, and the corresponding controls were transfected into the human alveolar epithelial cell line A549. Since stable cell cycle arrest is a key characteristic of cell senescence, we first analyzed the effect of the deficiency or upregulation of the enzyme on cell proliferation. As shown in Figure 5a, shMMP14 reduced by 95%, and +MMP14 increased by 90% the expression of the enzyme. Short hairpin control (shCtrl) and empty vector (Mock) do not affect. Overexpression of MMP14 induced a substantial increase in epithelial growth rate, while the silencing of MMP14 restricted it at 48 and 72 h (Figure 5b).

**Figure 6:**
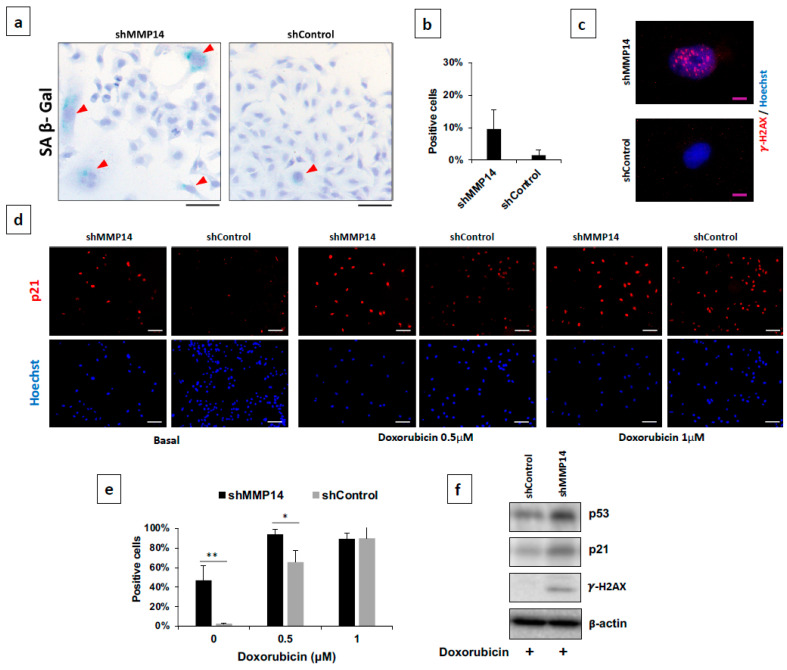
Silencing of MMP14 increases senescence markers on basal conditions and after treatment with doxorubicin. (**a**) Representative micrograph of senescence-associated β-galactosidase (SA-βGal) staining in shMMP14 cells (Red Arrowhead) versus shControl on basal conditions. Scale bar 100 μm. (**b**) Percentage of SA-βGal -positive cells. Graphic of two independent experiments. (**c**) Representative image of γH2AX foci in the nucleus of shMMP14 and shControl cells on basal conditions. Scale bar 10 μm. (**d**) Immunofluorescence of shMMP14 cells showing increased p21 on basal conditions and after stimulus with doxorubicin (0.5 or 1 μM) for 48h. Scale bar 100 μm. (**e**) Percent of P21 positive cells from two independent experiments. * *p* < 0.05, ** *p* < 0.01. (**f**) Western Blot of p21, p53, and γH2AX after doxorubicin 1 μM treatment of shMMP14 and shControl cells. Loading control was run on the same blot of p53.

Then, we examined canonical senescent markers in spontaneous and doxorubicin-induced senescence in MMP14-silenced A549 cells. We found that under basal conditions, an increased percentage of MMP14-silenced epithelial cells exhibited the typical marker senescence-associated beta-galactosidase (SA-βGal) activity that represents increased lysosomal β-galactosidase activity (Figure 6a,b). This observation was strengthened by the presence of γ-H2AX and the higher immunofluorescent expression of the cell cycle inhibitor p21 compared with shCtrl transfected epithelial cells (Figure 6c,d basal conditions).

Additionally, we used two concentrations of doxorubicin to stimulate A549 in order to amplify the senescence response and analyze the expression of other senescence-associated genes. Exposure of human epithelial cells to doxorubicin induced an increased number of p21 positive cells in MMP14-silenced cells (Figure 6d,e), and upregulation of γ-H2AX, p21 and p53 as shown by Western blot (Figure 6f). 

### 2.5. Senescent Epithelial Cells Overexpress Transforming Growth Factor-Beta (TGF-β) and Increase Profibrotic Markers in Fibroblasts

**Figure 7:**
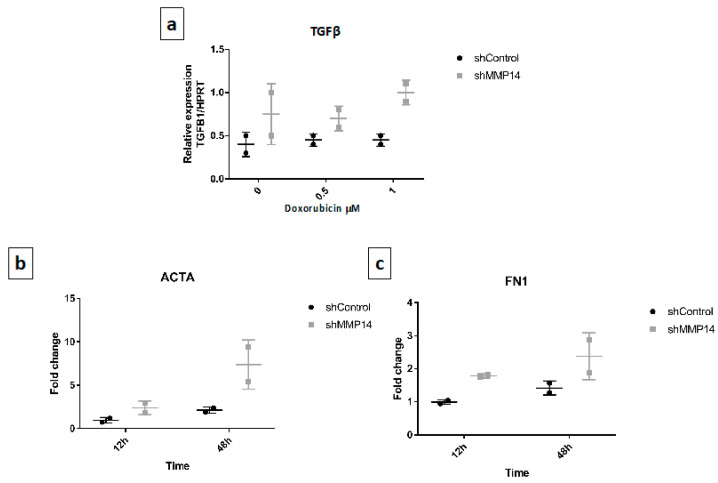
Senescent epithelial cells overexpress transforming growth factor-beta (TGF-β) and increase profibrotic markers in fibroblasts. (**a**) TFGβ1 expression of shMMP14 and control A549 epithelial cells on basal conditions and after doxorubicin (0.5 or 1 μM) treatment. (**b**,**c**). Effect of conditioned media (CM) of shMMP14 and control cells treated with doxorubicin 1 μM on normal lung fibroblasts. Fibroblasts were cultured for 12 and 48 h with 25% of A549 CM, and the expression of alpha-smooth muscle actin (ACTA) and Fibronectin1 (FN1) was examined by real-time polymerase chain reaction (RT-PCR). Graphics are representative of two lines of normal lung fibroblasts.

Since MMP-14 deficient-mice showed an increased lung fibrotic response and silenced epithelial cells in vitro showed a senescent phenotype we examined whether these senescent cells over expressed the prototypical profibrotic mediator transforming growth factor-beta (TGF-β). As shown in Figure 7a, under basal conditions and with doxorubicin MMP14-silenced cells showed increased expression of TGF-β over controls. 

Then we explored whether conditioned media of MMP-14-silenced epithelial senescent cells had an effect on human primary lung fibroblasts. We found that the expression of α-actin smooth muscle and fibronectin were upregulated (Figure 7b,c), although no effect was observed on α-1 type I collagen (data not shown).

## 3. Discussion

Idiopathic pulmonary fibrosis is a disease of unknown etiology characterized by an aberrant response of epithelial cells, that induce the formation of fibroblast/myofibroblast foci, followed by an excessive extracellular matrix accumulation and the progressive destruction of the lung parenchyma. In this context, understanding the molecular behavior of lung epithelium is important to reveal some of the pathogenic mechanisms implicated in the initiation and progression of IPF [3].

MMPs, a large family of endopeptidases, have emerged as critical regulators of lung homeostasis, and some of them may play an antifibrotic role while others are implicated in profibrotic processes [4]. MMP14 has been reported upregulated in lung epithelial cells examined by single-cell RNA sequencing [17] and specifically localized in alveolar epithelial cells of IPF patients by immunohistochemistry (IHC) [15]. Likewise, increased protein expression of MMP14 has been observed in epithelial cells of bleomycin-induced lung fibrosis [18,19]. These findings led us to consider that the epithelial expression of this enzyme could be involved in the biopathology of lung fibrosis.

Therefore, we generated a conditional mouse of Mmp14, which under doxycycline treatment presents a specific deletion of this enzyme in AEC2. These mice did not show any particular phenotype without lung challenge. Then, Mmp14 lung epithelial deficient mice were exposed to bleomycin injury to evaluate the role of this enzyme during the development of fibrosis and the period of resolution. 

Notably, we found that epithelial deletion of Mmp14 in the cKO mice resulted in exacerbated lung interstitial fibrosis and collagen accumulation after bleomycin exposure compared to control mice (WT and KO without doxycycline). Moreover, whereas the fibrotic response was largely resolved in the wild-type mice 120 days post bleomycin instillation, in Mmp14 deficient mice resolution overtime was impaired. We did not observe any difference in the phenotype of the triple transgenic mice before Doxycycline (DOX) treatment, and even after DOX in those animals that were not challenged with bleomycin. These data support that the difference in the fibrotic response is due to the loss of Mmp14. 

Similar results have been reported in mice with conditional knockout of Mmp14 specifically in stromal fibroblasts. Ablation of Mmp-14 in fibroblasts induced a fibrotic adult skin phenotype and furthermore, while dermal thickening decreased in WT mice after a resting period, this did not occur in Mmp14 cKO [20].

MMP14 belongs to a subfamily of membrane-associated metalloproteinases, and it has been reported as a key pericellular collagenase capable of degrading types I-III collagen fibers at the same site as the classic collagenases [21]. Moreover, mice deficient in Mmp14 has revealed that membrane-bound MT1-MMP is a powerful collagenase, necessary for cell invasion through collagen-rich extracellular matrices [22]. Therefore, the deficiency of this enzyme could contribute to aggravate the fibrotic response and to delay the time of resolution through a lower rate of collagen degradation. However, this effect should be more relevant when the deficiency affects myofibroblasts as was shown in skin fibrosis where MMP-14-deficient fibroblasts lost their ability to process fibrillar collagen type I and to activate proMMP-2 [20]. In contrast, the effects of Mmp14 deficiency specifically in the lung epithelium on the fibrotic response was unknown and less plausible to be related to the interstitial collagenolytic activity.

In this context, it has been demonstrated that the abnormal proteolytic processing of ECM that occurs in Mmp14-deficient mice triggers signaling pathways that induce cellular senescence through severe alterations in the nuclear architecture and the cytoskeleton [23]. Moreover, when Mmp14-mediated proteolytic activity is increased, the induction of the senescence program is attenuated providing additional evidence that abnormal ECM remodeling may lead to cellular senescence [23]. 

In the last few years, a strong body of evidence indicates that aberrant accumulation of epithelial senescent cells, a hallmark of aging, is associated with the worsening of fibrosis and other detrimental effects on lung homeostasis [24]. Cellular senescence is characterized by a prolonged and essentially irreversible cell-cycle arrest due to the upregulation of cell-cycle inhibitors, and high activity of senescence-associated β-galactosidase. Senescent cells secrete a plethora of factors, including pro-inflammatory cytokines and chemokines, growth modulators, angiogenic factors, and MMPs, causing profound changes in the local microenvironment [25].

On these bases, we performed gain/loss experiments over-expressing or silencing MMP14 in the human type 2 AEC A549 to evaluate cell senescence. We did not use primary AEC2 from mice lungs because they do not proliferate in vitro, and thus these cells were isolated only to corroborate the specific Mmp14 gene deletion.

Successful MMP14 knockdown via RNA silencing in A549 cells led to epithelial cell senescence characterized by increased SA-β-Gal activity and accumulation of γ-H2AX, a DNA damage marker. p53 and p21 were also upregulated in MMP14 deficient A549 cells. It is well known that p53 activation by the DNA damage signaling cascade is a critical step in the induction of cellular senescence. 

One common feature of senescent cells is an irreversible cell-cycle arrest. Cyclin-dependent kinase inhibitor 1A (CDKN1A, also known as p21) is critical during the induction of p53-dependent cellular senescence arresting cell cycle mainly during the G1/S-phase, and it was also upregulated in the MMP14 deficient A549 cells [25,26]**.** Supporting these findings, MMP14 deficient A549 cells showed a marked decrease in cell proliferation while a strong increase was observed in the cells transfected with the enzyme. It is well known that senescent cells are metabolically active and can affect their microenvironment through their senescence-associated secretory phenotype, resulting in multiple physiological or pathological processes. Finally, linking the senescent phenotype of epithelial cells in vitro with the increased fibrotic response in vivo, we found that these senescent cells showed an increased expression of TGF-β1 and stimulated fibroblasts for the expression of known profibrotic molecules such as αSMA, suggesting fibroblast to myofibroblast differentiation, and the ECM fibronectin. 

In summary, the current study demonstrated that epithelial deficiency of MMP14 exacerbates bleomycin-induced experimental lung fibrosis and impair remodeling resolution. The mechanisms appear to be associated with the induction of an epithelial senescence program.

## 4. Material and Methods

### 4.1. Human Samples

The study protocol for tissue donation from patients was conducted according to the relevant guidelines and regulations. Informed consent was obtained for all participants. This protocol was approved by the Bioethics Committee of the Instituto Nacional de Enfermedades Respiratorias Ismael Cosío Villegas (INER) in Mexico City (Identification code: B-036: Date September, 2015). Tissue from 4 patients of IPF and two controls was used. IPF diagnosis was undertaken by the Interstitial Lung Disease Program of the INER according to the ATS/ERS/ALAT guidelines. 

### 4.2. Immunohistochemistry (IHC)

IHC was performed as previously described [27]. Briefly, lung sections were deparaffinized, rehydrated, and then blocked with 0.45% H_2_O_2_ in methanol for 30 min followed by normal serum (Vector Laboratories, Burlingame, CA, USA) diluted 1:20 in PBS for 20 min. After, antigen retrieval with citrate buffer was performed. The primary antibody for MMP-14 (GeneTex, GTX54361, Irvine, CA, USA was applied and incubated at 4 °C overnight. A secondary biotinylated anti-immunoglobulin followed by horseradish peroxidase-conjugated streptavidin (BioGenex, San Ramon, CA, USA) was used according to the manufacturer’s instructions. 3-Amino-9-ethyl carbazole (BioGenex) in acetate buffer containing 0.05% H_2_O_2_ was used as the substrate. The sections were counterstained with hematoxylin. The primary antibody was replaced by non-immune serum for negative control slides.

### 4.3. Mice

Animal use complied with the relevant guidelines and regulations for Animal Care and the protocol was approved by the Bioethics Committee of INER in Mexico City (Identification code: B-036: Date September, 2015). Sftpc-Mmp14-cKO mice were generated from Mmp14^Fl/Fl^ mouse and Sftpc^(rtTA^/^TetO-Cre)^ [28] mouse kindly donated by Carlos López-Otín and Ana L. Mora respectively. C57BL6/129SvJ mice carrying the three transgenes (rtTA/TetO-Cre/MMP14Fl) made the deletion of Mmp14 possible after doxycycline treatment (100 mg/L in the sterile water with 50% sucrose) administered from the 6^th^ week of age and continued for the whole life of the animals (Figure 2a).

Sftpc-Mmp14-cKO mice with and without doxycycline stimulus were treated with an intratracheal injection of 25 µL of phosphate-buffered saline (PBS) or bleomycin (0.05 U/10 g) and sacrificed at 21, and 120 days. Bleomycin instillation was performed in the 8th week of age. Right lungs were fixed with 4% paraformaldehyde for staining with hematoxylin and eosin, and Masson’s trichrome stains. Lung sections were coded and scored blindly for severity and extend of the fibrotic lesions as previously described [29]. 

### 4.4. Colorimetric Determination of Hydroxyproline

Left lungs from mice were hydrolyzed in 6 N HCl for 24 h at 110 °C. Hydroxyproline was determined by measuring the hydroxyproline content as previously described [30]. Briefly, Aliquots were assayed by adding chloramine T (Sigma-Aldrich, St Louis, MO, USA) solution followed by development with Erlich reagent. Each sample was tested in triplicate. Data are expressed as mg of hydroxyproline/left lung.

### 4.5. Epithelial Cells Isolation 

Alveolar epithelial cells were sorted using a positive selection of Anti-CD326/EpCam magnetic microbeads (Miltenyi Biotec, 130-105-958, Bergisch Gladbach, Germany). Briefly, lungs were subjected to manual dissection and were perfused with dispase (Corning) and DNase (50 mg/mL; Sigma-Aldrich) to obtain single-cell suspensions. Then, the cell suspension is loaded onto a MACS® Column which is placed in the magnetic field of a separator (Miltenyi Biotec, MultiMACS Cell 24 Separator Plus).

### 4.6. Cell Culture 

Human lung epithelial cell line A549 was purchased from ATCC (Manassas, VA, USA). Cells were maintained in an incubator with a humidified environment containing 95% fresh air and 5% CO_2_ at 37 °C in nutrient mixture F-12 (Invitrogen, Carlsbad, CA, USA), supplemented with 5% FBS (Gibco, Waltham, MA, USA), 100 units/mL penicillin, 100 μg/mL streptomycin. Primary lung fibroblasts were obtained from normal tissues as previously described [31].

### 4.7. Cell Transfection 

Full-length human MMP14 cDNA cloned into the pCMV6-AC-GFP vector was obtained from Origene (RG208917). MMP14 was silenced by the overexpression of short hairpin RNA directed to MMP14. 

### 4.8. Western Blot 

Cells were lysed using radioimmunoprecipitation assay buffer (RIPA), (Sigma, Saint Louis, MO, USA), protein extract was used for electrophoresis in a polyacrylamide gel. After, proteins were transferred to PVDF membranes and blocked for 1 h. Primary antibodies were incubated overnight MMP14 (GeneTex, GTX543611:1000), p53 (R&D, AF1355, 1:2000, Minneapolis, MN, USA), p21 (Santa Cruz, sc-6246, 1:400 Santa Cruz, CA, USA) γH2AX (Abcam, ab81299 phosphorylation of S139, 1:500, Cambridge, MA, USA). HRP-secondary antibodies were used, and membranes were developed in a ChemiDoc (BioRad, Hercules, CA, USA).

### 4.9. Growth Rate Assay 

A549 cells overexpressing and silenced for the enzyme were seeded in 96-well plates, by triplicate in 4 independent experiments, as well as the respective controls. WST-1 (water-soluble tetrazolium salt) reagent was used according to the manufacturer recommendations.

### 4.10. β-Galactosidase Assay

Cells were set on coverslips, fixed, and stained with the β-galactosidase activity kit according to the manufacturer’s instructions (Merck, Kenilworth, NJ, USA). Nuclei were counterstained with hematoxylin. Positive cells were determined by counting β-galactosidase-positive cells over total cells per field.

### 4.11. Real-Time Polymerase Chairn Reaction (PCR)

Total RNA was extracted with Trizol reagent (Invitrogen), according to the manufacturer’s recommendations. 1 μg of RNA was reverse transcribed into cDNA using Verso cDNA Synthesis Kit (Applied Biosystems). For mice expression of Mmp14, Sftpc, and Gapdh, as well as for epithelial expression of MMP14 and hypoxanthine phosphoribosyltransferase 1 (HPRT), Taqman probes were used, respectively: Mm00485054_m1, Mm00488144_m1, Mm99999915_g1, Hs01037003_g1, and Hs01037003_g1. 

Moreover, several genes were studied with SYBR Green PCR Master Mix (Thermo, Waltham, MA, USA): collagen type I alpha 1 chain (COL1A1), fibronectin containing extra domain A (FnEDA), alpha-smooth muscle actin 2 (ACTA2), transforming growth factor-beta 1 (TGFB1), HPRT and succinate dehydrogenase (SDH). Oligonucleotides used are shown in Table 1.

### 4.12. Immunofluorescence

Cells were cultured in coverslips, fixed with 4% of paraformaldehyde for 15 min, and permeabilized with Triton x-100 (Research Organics, Cleveland, OH, USA). The antibody incubation was conducted overnight at 4 °C with the following antibodies: p21 (Santa Cruz, sc-817, 1:50) and γH2AX (Abcam, ab81299 phosphorylation of S139, 1:100). After the secondary antibody (Donkey anti-Mouse Alexa Fluor 594 Invitrogen, Catalog # A-21203) was incubated for 1 h at room temperature, nuclei were stained with Hoechst (Thermo). Samples were then mounted and imaged in ZOE™ Fluorescent Cell Imager (Bio-Rad). Quantification was performed with ImageJ software (NIH). 

### 4.13. Statistical Analysis

All data are expressed as mean ± standard deviation (SD). Two-tailed Student *t*-test and one-way ANOVA with Tukey post hoc were used. All statistical analyses were performed using GraphPad Prism version 7.00 (La Jolla, CA, USA). 

## Figures and Tables

**Table 1:** Oligonucleotides used in real-time PCR.

Gene	Direction	Sequence (5′ --> 3′)
COL1A1	Forward	GAGGGCCAAGACGAAGACATC
	Reverse	CAGATCACGTCATCGCACAAC
FnEDA	Forward	CTGGTTCAGACTGCAGTAACC
	Reverse	CAGGGAATAGCTCATGGATTCC
ACTA2	Forward	AAAAGACAGCTACGTGGGTGA
	Reverse	GCCATGTTCTATCGGGTACTTC
TGFB1	Forward	GGCCAGATCCTGTCCAAGC
	Reverse	GTGGGTTTCCACCATTAGCAC
HPRT	Forward	AAGGACCCCACGAAGTGTTG
	Reverse	GGCTTTGTATTTTGCTTTTCCA
SDH	Forward	CAAACAGGAACCCGAGGTTTT
	Reverse	CAGCTTGGTAACACATGCTGTAT

## Data Availability

Data is contained within the article.
